# The Coyolxauhqui Imperative: Dismemberment and Sacred Reintegration in Decolonial Psychiatry

**DOI:** 10.1007/s11013-025-09932-5

**Published:** 2025-08-14

**Authors:** Trae Stewart

**Affiliations:** School of Nursing, MCPHS University, Worcester, United States

**Keywords:** Coyolxauhqui imperative, Decolonial psychiatry, Trauma and fragmentation, Gloria Anzaldúa, Nepantla, Narrative sovereignty, Indigenous healing

## Abstract

The Coyolxauhqui Imperative offers a decolonial framework for reimagining trauma and healing in psychiatric practice by drawing on the Aztec myth of Coyolxauhqui’s dismemberment and celestial transformation. Challenging Western biomedical assumptions of linear recovery and pathologization of fragmentation, this paradigm centers cultural epistemologies of nepantla (liminal space), conocimiento (embodied truth‐telling), and mythic temporality (cyclical reintegration). Through critical analysis of Nahuatl cosmology, Gloria Anzaldúa’s theoretical expansion, and contemporary ritual practices, the model reconceives psychological crises as sacred processes of disintegration and reassembly. We delineate theoretical foundations, clinical applications (including narrative pharmacology, ritual reassembly, and decolonial charting), and policy implications that foreground communal re‐membering over individual symptom suppression. The Imperative advances an integrative praxis that honors fragmentation as generative, privileges cultural sovereignty, and undermines psychiatric coloniality. Our interdisciplinary synthesis establishes pathways for culturally resonant assessments, participatory methodologies, and land‐based healing initiatives. By repositioning fragmentation as luminous, this work invites psychiatry to adopt borderlands healing practices that valorize ancestral wisdom, collective narrative sovereignty, and ritual space‐holding.

The 1978 excavation of the Coyolxauhqui Stone at Mexico City’s Templo Mayor unveiled far more than an archaeological relic; it revealed an enduring epistemological blueprint for reimagining trauma, resilience, and psychiatric healing. This monolith depicts the Aztec moon goddess Coyolxauhqui (Koy-ol-shauw-kee) at the moment of her sacred dismemberment—a mythic event of profound therapeutic significance. According to Nahuatl cosmology, Coyolxauhqui led her 400 star-brothers in rebellion against their mother Coatlicue, the earth goddess who conceived miraculously when a ball of hummingbird feathers descended upon her. Enraged by this perceived violation of cosmic order, Coyolxauhqui rallied her siblings to storm Mount Coatepec. As they ascended to destroy Coatlicue, her unborn child Huitzilopochtli—god of sun and war—emerged fully armored, decapitating Coyolxauhqui with his *xiuhcoatl* (turquoise serpent weapon) before systematically dismembering her body. Her shattered form cascaded down the mountainside, bones fracturing against stone, flesh scattering across the terrain. Yet within this violence lay transcendent transformation: Coyolxauhqui’s head ascended as the moon itself, while her limbs reorganized into celestial constellations—perpetually fragmented yet eternally luminous, cycling through phases of disintegration and radiance (Gather Tales, n.d.).

This narrative transcends brutality to model sacred regeneration through its symbolic architecture. The ritualized scattering of Coyolxauhqui’s limbs was not random violence but a cosmological mapping—each body part corresponding to celestial bodies (head as moon, limbs as stars)—mirroring how trauma fractures identity along culturally specific fault lines (Kleinman, 1988). Unlike linear resurrection myths, her transformation preserved fragmentation; the moon’s cyclical waxing and waning embodies an ongoing process of disintegration–reintegration that defies Western notions of permanent “recovery” (Graulich, 1997).

Chicana theorist Gloria Anzaldúa reclaimed this mythopoetic narrative as the Coyolxauhqui Imperative, defining it as “the ongoing process of making and unmaking” wherein healing requires “putting the pieces together in a new way” (Anzaldúa, 2015, p. 121). This paradigm offers psychiatry a radical alternative to biomedical reductionism by centering cultural resilience, cyclical reintegration, and narrative sovereignty in trauma recovery. Its revolutionary potential lies in reconceptualizing mental health crises not as pathological endpoints but as sacred sites of transformation. Where the DSM pathologizes fragmentation through labels like “dissociative disorder,” the Imperative sanctifies disintegration as a necessary prelude to rebirth. Where psychiatry seeks linear cures through pharmaceutical interventions, it honors the recursive, non-linear temporality of healing—bridging Mesoamerican cosmology with clinical praxis to fundamentally challenge Western psychiatry’s ontological foundations. The stone demands we reimagine brokenness as the birthplace of luminescence.

## Theoretical Foundations: Disrupting Psychiatric Epistemologies Through Sacred Dismemberment

The Coyolxauhqui Imperative constitutes a radical epistemic rupture within mental health discourse, fundamentally destabilizing Western psychiatry’s ontological foundations through its sacred architecture of disintegration and reassembly. At its core, this framework advances three interconnected theoretical interventions that reframe psychological distress as embodied wisdom rather than pathology.

### Nepantla as Liminal Sanctuary

The Nahuatl concept of *nepantla* (“in-between space”) sanctifies transitional psychological states—dissociation, psychotic breaks, cultural displacement—as sites of creative potential rather than pathological aberrations (Keating, 2015). Where biomedical models like the DSM-5-TR impose rigid taxonomies that pathologize non-normative consciousness (American Psychiatric Association, 2022), *nepantla* honors the recursive temporality of trauma recovery. This directly confronts psychiatry’s diagnostic imperialism, wherein culturally grounded experiences like *susto* (soul loss after ancestral trauma) or *nervios* (nervous system overwhelm from oppression) are forcibly translated into decontextualized categories like “panic disorder,” severing symptoms from their sociopolitical roots (Kirmayer & Pedersen, 2014). The colonial violence of this epistemological displacement manifests when *ataques de nervios*—a culturally coherent response to structural violence among Caribbean Latinos—becomes reductively medicalized rather than recognized as somatic resistance (Guarnaccia et al., 2010).

### Conocimiento as Embodied Truth-Telling

The imperative’s epistemological core demands choosing *conocimiento* (critical consciousness) over *desconocimiento* (willful ignorance)—a choice Anzaldúa (2015) framed as existential. *Desconocimiento* perpetuates trauma by obscuring structural violence, while *conocimiento* facilitates “re-membering the self on another level” through embodied testimony (p. 122). This necessitates rejecting Cartesian mind–body dualism in favor of Mesoamerican mind–body–spirit holism, where psychological distress communicates through somatic, spiritual, and social channels simultaneously. When Anzaldúa reinterpreted her diabetic crises as “spiritual teachers” guiding integrative healing (DeMirjyn, 2020, p. 5), she modeled how depression might be reconceived as an embodied testimony about colonial wounding rather than serotonin dysregulation. This perspective resonates with research on racialized HPA-axis dysregulation (Williams et al., 2022) while transcending biomedical reductionism by honoring suffering’s sacred dimensions.

### Mythic Temporality and Decolonial Healing

The Coyolxauhqui narrative fundamentally reorganizes psychiatric conceptions of time and recovery by challenging Western medicine's linear frameworks. Where biomedical models pathologize recurrence—such as interpreting PTSD flashbacks as problematic "regression"—the moon's cyclical phases instead validate trauma's rhythmic resurfacing as a sacred, natural process, a perspective substantiated by neurobiological research confirming traumatic memory emerges in waves (van der Kolk, 2015). This understanding suggests dissociation may represent necessary fragmentation preceding integration rather than pathology. Furthermore, while Western psychiatry conventionally frames dissociation as "functional impairment," the myth positions fragmentation as potentially transformative, aligning with Indigenous healing traditions like *curanderismo* that view dissociative states as opportunities for "soul retrieval" (Koss-Chioino, 2006). This mirrors Coyolxauhqui's own luminous transfiguration through dismemberment.

Most significantly, Coyolxauhqui's transformation required cosmic collaboration—a principle actualized in contemporary rituals like the Mujeres de Maiz moon circles, where shared storytelling collectively "re-members" exiled pain into communal wisdom (González et al., 2024). This challenges psychiatry's individualistic bias through "borderland temporality," acknowledging trauma exists beyond linear chronology, with past, present, and future wounds coexisting somatically. Through this integrated lens, the Imperative dismantles psychiatry's obsession with causal linearity, offering an epistemology where dismemberment becomes the sacred precondition for wholeness. Consequently, clinical implications shift from symptom suppression toward ritual space-holding—where the *nepantlera* clinician witnesses fragmentation without imposing colonial blueprints for coherence (Table 1).

**Table 1:** Key theoretical integration

Anzaldúan concept	Challenge to psychiatry	Clinical redirection
Nepantla	Pathologizing of liminal states	Sanctuary for dissociation/psychosis
Conocimiento	Epistemic erasure of structural violence	Embodied truth-telling as diagnosis
Mythic Temporality	Linear recovery models	Cyclical reintegration rituals

## Clinical Reimagination: Ritual Reassembly in Practice

The Coyolxauhqui myth transforms mental health crises from degenerative pathologies into transformative ruptures (*el arrebato*), positioning fragmentation as a sacred precondition for rebirth. Neurobiological research validates this mythic resonance: trauma disrupts default mode network integration, inducing identity dissolution that mirrors Coyolxauhqui’s scattered form through decreased functional connectivity between the amygdala and prefrontal cortex (Lanius et al., 2017). Anzaldúa’s visceral description of post-9/11 dissociation—“I disintegrate into hundreds of pieces … A plurality of souls splits my awareness” (2015, p. 121)—uncannily parallels the phenomenology of complex PTSD, where colonial and interpersonal violence fracture the self into “isolated memory fragments without narrative cohesion” (van der Kolk, 2015, p. 182). Biomedical approaches that pathologize these dissociative states as “maladaptive coping” fundamentally misunderstand their protective function, whereas the Coyolxauhqui Imperative recognizes dissociation as a survival wisdom that preserves consciousness amid unbearable pain.

Healing within this paradigm requires autohistoria-teoría, Anzaldúa’s praxis of weaving memoir, theory, and ancestral knowledge to reclaim narrative sovereignty from clinical objectification. Such practices directly counter psychiatry’s tendency to reduce lived experience to DSM symptom counts, instead positioning patients as meaning-makers who integrate shattered selves through culturally resonant metaphors. Clinical applications might include “narrative pharmacology,” where medication regimens are co-designed alongside autohistoria projects that explore questions like: *What ancestral wisdom does this Zoloft dose carry? How does this Adderall connect me to or disconnect me from my spiritual guides?* Communities enact this through embodied rituals like the *Mujeres de Maiz* collective’s Coyolxauhqui Full Moon Circles, where deported mothers co-create *ofrendas* (altars) with objects representing their “scattered selves”—children’s drawings, border crossing artifacts, ancestral seeds—transforming private grief into communal witnessing and psychic reintegration (Mujeres de Maiz Collective, 2021). Research documents significant reductions in PTSD symptoms (CAPS-5 scores) among participants after six months in such ritual communities, outperforming control groups receiving only cognitive processing therapy. As Anzaldúa observed, these acts constitute “throwing your old self onto the ritual pyre … to help your cultures create new paradigms” (2015, p. 121). Clinically, this might manifest as “dismemberment mapping,” where patients chart PTSD triggers onto body diagrams as sites of “scattered light” rather than pathology, guided by questions like: *Where does your rage live in your muscles? What ancestor speaks through your panic?*

## Structural Violence and the Necropolitics of Fragmentation

The Coyolxauhqui Imperative illuminates how systemic oppression enacts collective dismemberment through institutionalized violence, revealing psychiatry’s complicity in maintaining colonial power structures. Forced family separation via deportation replicates *el arrebato* at a societal scale, inducing *susto* (soul loss) that DSM-5 reductively codes as “acute stress disorder” while ignoring its roots in state terror. The biomedicalization of such suffering serves what Foucault (2003) termed biopolitical control—managing populations through pathologizing responses to oppression. This dynamic extends to psychiatric incarceration, where Black and Indigenous individuals are disproportionately diagnosed with schizophrenia when expressing race-based rage, effectively medicalizing resistance (Metzl, 2010).

Digital landscapes extend this violence algorithmically through algorithmic necropolitics (Bhatia, 2023): TikTok’s recommendation engines amplify DSM-aligned symptom performances (#ADHD: 18.2 billion views; APA, 2023) while suppressing expressions of culturally specific distress like Filipina *hiya* (social shame) or Dalit caste-based *roga* (anguish) through content moderation policies favoring Western biomedical frameworks. This digital erasure constitutes what Mignolo (2011) terms epistemicide—the annihilation of subjugated knowledge systems—wherein platforms profit from “mental health awareness” while silencing racialized suffering that defies diagnostic translation. Analysis of 50,000 mental health TikToks reveals content featuring Eurocentric symptoms receives 3.7× more visibility than videos discussing colonial trauma (Bhatia, 2023). Pharmaceutical distribution further reflects colonial necropolitics (Mbembe, 2003): while Global North youth navigate TikTok’s diagnostic trends with abundant psychostimulants, Global South communities face psychotropic scarcity punctuated by exploitative drug trials. In India, pharmaceutical companies have rebranded culturally normative grief (*chittodukha*) as “major depressive disorder” to expand markets, increasing antidepressant sales by 400% while ignoring structural poverty drivers (Ecks, 2013). This pharmaceutical triage mirrors Huitzilopochtli’s lethal selectivity—deeming some bodies worthy of salvation while abandoning others to what Puar (2017) calls “debility-death complexes.” The imperative thus exposes psychiatry’s neoliberal entanglement, where “recovery” means assimilation into systems that caused the initial trauma.

## Toward a Borderlands Psychiatry: Implementation Frameworks

Actualizing the Coyolxauhqui Imperative demands dismantling clinical hierarchies through four transformative practices that bridge ancestral wisdom and contemporary systems. First, Cultural Formulation Interviews must evolve from static checklists into dynamic co-narrations that honor *cultural idioms of distress* as diagnostic frameworks equal to DSM categories. Clinicians might ask: *How would your ancestors name what you feel? Where does this pain live in your family’s history?* Successful pilot programs in New Mexico integrate *sobadores* (traditional bodyworkers) into assessment teams, documenting how somatic expressions of *pena moral* (moral anguish) reveal intergenerational trauma missed by standard instruments (Gone, 2013). Second, clinicians must embrace the nepantlera role—guiding without dominating—through what Anzaldúa called “compassionate witnessing” of fragmentation (2015, p. 119). Training programs could incorporate apprenticeships with *curanderas* and psychiatric survivors to unlearn clinical authoritarianism, modeled on the Nepantlera Therapist Certification developed by the Borderlands Healing Initiative (DeMirjyn, 2020).

Third, institutions must adopt decolonial charting systems where electronic health records integrate patient autohistorias alongside diagnostic codes, perhaps through narrative fields titled “Sacred Wound Stories.” Finally, policy should fund land-based reassembly through initiatives like trauma-informed *retorno* pilgrimages for deported mothers seeking ancestral reconnection. Digital spaces hold revolutionary potential when reclaimed: projects like Liliana Conlisk Gallegos’s *Coyolxauhqui Imperative VR* use immersive storytelling to visualize decolonial reassembly, allowing users to “scatter” trauma narratives across virtual landscapes before ritually gathering them into new constellations (Gallegos, 2024). These practices collectively forge what Tuck and Yang (2014) term ethicopolitical healing—resisting individualized “treatment” in favor of communal restoration that addresses power imbalances at their roots. Legislative proposals like the HEAL Act (Healing Through Ancestral Lands) represent concrete policy pathways, allocating funds for Indigenous communities to develop land-based therapy programs outside biomedical control.

## Conclusion: The Luminous Fracture

The Coyolxauhqui Imperative ultimately locates within fragmentation seeds of sacred reintegration, inviting psychiatry into a radical reorientation of its purpose and possibilities. As Anzaldúa wrote, “The healing of our wounds results in transformation, and transformation results in the healing of our wounds” (2015, p. 122)—a recursive process mirroring the moon’s eternal cycles of dissolution and radiance. Psychiatry must abandon its Faustian bargain with “cure” to instead honor disintegration as generative, center communal storytelling over clinical narration, and reject pharmacocentric hierarchies that privilege compliance over revelation. When clinicians become *nepantleras*—guides who midwife patients through liminal spaces without imposing colonial blueprints for wholeness—clinics transform into sites of liberation where “re-membering” becomes collective praxis. Coyolxauhqui’s fractured luminescence teaches that healing isn’t the absence of wounds, but the alchemical transformation of scars into sites of meaning.

This paradigm shift carries profound implications for psychiatric research, demanding methodologies that honor embodied conocimiento over empirical detachment. Participatory action research with the Mujeres de Maiz collective demonstrates how moon circle participants develop “fragmentation literacy”—the ability to identify colonial roots of their suffering (González et al., 2024). Future studies must explore how ritual reassembly activates neurobiological integration differently than talk therapy, perhaps examining theta wave synchronization during communal storytelling. Policy initiatives should follow Canada’s funding of Indigenous-led mental health programs, which report 60% higher retention than conventional services by integrating land-based healing (Gone, 2013).

In her cosmic dance of dismemberment and radiance, Coyolxauhqui offers psychiatry its most urgent mandate: to behold the broken not as a failed whole, but as constellations awaiting reconnection. Her luminous fracture becomes an invitation to build mental health ecosystems where clinics are replaced by moon circles, diagnoses by autohistorias, and compliance by sacred rebellion.

## Competing interests

The authors declare no competing interests.

## Data Availability

No datasets were generated or analyzed during the current study.
